# Lésions concomitantes aux genoux flottants et gravite

**DOI:** 10.11604/pamj.2016.25.83.7920

**Published:** 2016-10-17

**Authors:** Daniel Handy Eone, Léopold Lamah, Jean Emile Bayiha, Danielle Larissa Essomba Ondoa, Bernadette Ngo Nonga, Farikou Ibrahima, Jean Bahebeck

**Affiliations:** 1 Service de Chirurgie Orthopédique et de Traumatologie de l’Appareil Moteur (S.C.O.T.A.M) Hôpital Central de Yaoundé, Cameroun; 2 Service d’Orthopédie - Traumatologie CHU, Yaoundé, Cameroun; 3 Service de Chirurgie CHU Yaoundé, Cameroun; 4 Centre National des Handicapés Moteurs Yaoundé, Cameroun

**Keywords:** Genou flottant, polytraumatisme, lésion associée, Floating knee, polytraumatism, associated lesion

## Abstract

Le genou flottant est issu d’un traumatisme de haute énergie, dont la genèse suggère de vaste dégâts aussi bien sur le plan locorégional que général. Faisant référence au polytaumatisme. Le but de notre étude était de recenser toutes les lésions concomitantes au genou flottant dans notre milieu de pratique et d’évaluer la sévérité. Nous avons mené une étude descriptive et rétrospective couvrant une période de 14 ans et 9 mois. Sur un échantillon de 75 genoux flottants, avec une moyenne d’âge de 35 ans. Soixante six patients avaient obtenu un score d’ISS supérieur ou égal à 16 donc qualifié de polytraumatisé. Les traumatismes crâniens, les lésions thoraciques et abdominales retrouvés en même temps que le genou flottant exigent une réanimation adéquate.

## Abstract (en)

Floating knee is caused by high-energy trauma, whose genesis is suggestive of extensive locoregional and general damages. Referring to multiple trauma. The aim of our study was to collect data on all concomitant floating knee injuries in our practice environment and to evaluate their severity. We conducted a descriptive and retrospective study over a period of 14 years and 9 months. Our sample consisted of 75 floating knees, the average age was 35 years. Sixty six patients had an ISS≥16 (classified as polytrauma). Head traumas, chest and abdominal injuries associated with floating knee injuries require adequate resuscitation.

## Introduction

La fracture simultanée et homolatérale du fémur et du tibia, est issue d’un traumatisme de haute énergie [1]. Dans ce contexte il, n’est pas surprenant que les forces ayant été à l’origine de ces fractures aient crées des lésions concomitantes dans le corps de ces patients. Ces derniers sont souvent en état de choc hémodynamique, et ont besoin d’être réanimé à la phase initiale de leur prise en charge. Le Mécanisme lésionnel communément retrouvé est une collision véhicule contre moto lors d’un dépassement [2]. La genèse de cette lésion suggère de vaste dégâts aussi bien sur le plan local que général; donc se référent au tableau de polytraumatisme. Toute fois le membre grossièrement tuméfié que l’on rencontre dans le genou flottant agit comme un facteur majeur de distraction responsable d’une évaluation erronée de la gravité des lésions. Ainsi il devient nécessaire de réaliser une évaluation initiale approfondie et minutieuse de l’étendue, de la complexité et de la gravité des lésions. Le but de ce travail était de recenser toutes les lésions concomitantes au genou flottant dans notre milieu et d évaluer la sévérité.

## Méthodes

Il s’agissait d’une étude descriptive rétrospective couvrant une période de 14 ans et 9mois. (Janvier 2000 à Octobre 2014) qui s’est tenue dans le service de chirurgie orthopédique et de traumatologie de l’appareil moteur (SCOTAM) de l’hôpital central de Yaoundé après une approbation du comité éthique de la dite institution hospitalière. Cette étude a porté un intérêt sur les patients adultes âgés de 16 ans et plus. Des deux sexes admis au service pour genou flottant, possédant un dossier médical complet. Nous nous sommes servis de variables telles que: Le sexe, l’âge, la profession, un bilan radiographique complet, le siège du genou flottant, et le type selon la classification de FRAZER [3], l’existence ou non de lésions concomitantes (locales, locorégionales, générales) et du score de sévérité lésionnel (Injury Severity score) défini par BAKER en 1974. Nous a permis d’apprécier la sévérité des lésions.

## Résultats

Nous avons colligé un total de 75 genoux flottant dont deux atteintes bilatérales. Notre série était constituée d’une population à prédominance masculine dont l’âge varie entre 16 et 81 ans avec une moyenne de 35 ans. La tranche d’âge la plus atteinte était celle comprise entre 25 et 32 ans. Soit 72%. Une proportion de 51% de sujet exerçait des emplois appartenant au secteur informel parmi lesquels 20% étaient des agriculteurs. Dans 90% des cas, l’étiologie du genou flottant était un accident de la circulation. Les autres étant essentiellement représentés par les accidents d’abattage, les chutes d’un lieu élevé et des plaies par armes à feu. Les fractures intéressaient le membre inférieur gauche dans 73% des cas et dans 02 cas soit 2% l’atteinte était bilatérale. Cinquante deux genoux flottant (70 %) étaient classés selon FRAZER et repartis tel que suit (Tableau 1): soit une implication articulaire estimé à 44%. Cinquante six genoux flottants (75%) possédaient au moins une ouverture proximale et ou en distale. Selon la classification de Gustillo et ANDERSON, (Tableau 2) Ce qui reflète un important délabrement cutané suite au traumatisme au niveau local. Cinquante un malades (68%) sur un total de soixante treize patients ont présenté des lésions concomitantes et dans 70,60% des cas elles étaient homolatérales au genou flottant. Les lésions concomitantes quand à elles (Tableau 3). Un patient suite à une plaie par arme à feu, a présenté une atteinte de l’artère poplitée cliniquement diagnostique et vu après 06 heures de temps. Par ailleurs aucune lésion nerveuse ni ligamentaire n’a été mentionné dans nos registres. La gravité de ces lésions a été évaluée en utilisant l’ISS. Le score moyen était de 21. Soixante six malades soit 90% avaient obtenu un score supérieure ou égal à 16, et par conséquent qualifié de polytraumatisés.

**Tableau 1:** Répartition des genoux flottants selon la classification de FRAZER

Type	Nombre
Type I	26
Type IIa	10
Type IIb	09
Type IIc	07
*TOTAL*	52

**Tableau 2:** Classification des lésions cutanées selon Gustillo et Anderson

TYPE	CUISSE	JAMBE	TOTAL
I	—	08	*08*
II	11	12	*23*
IIIa	06	06	*12*
IIIb	04	04	*08*
IIIc	02	03	*05*
**TOTAL**	*23*	*33*	**56**

**Tableau 3:** Répartition topographique des lésions concomitantes

Topographique	Nombre	Lésions cliniques	
Tête et cou	**23**	Traumatisme Crânien	12
		Traumatisme Maxillo-faciale	08
		Traumatisme Oculaire	03
Thorax	**07**	Fracture de côtes	05
		Contusion Thoracique	02
Abdomen	**08**	Rupture de viscère pleins	05
		Contusion sans lésion d’organe	03
Ceinture pelvienne	**07**	Fracture du Bassin	05
		Lésions Uro- Génitales	02
Membre Supérieur	**08**	Fractures	06
		Entorses et contusion Musculaires	02
Membre Inférieur	**22**	Fractures	20
		Entorse et Contusion musculaire	02

## Discussion

Notre série rapporte 75 genoux flottants répertoriés chez 73 patients. La prédominance masculine, la moyenne d’âge de 35 ans et la tranche d’âge la plus atteinte ainsi que deux cas de genoux flottants bilatéral. Divers séries Africaines ayant traité ce sujet, malgré la taille variable des séries, [4–6] cette lésion atteint l’adulte masculin jeune et actif. Le secteur d’activité également est un facteur non négligeable. Car les patients exerçants dans le secteur informel se déplacent très souvent par la un engin à deux roues. Car peu couteux et ont accès dans les zones où les véhicules n’arrive pas. Hans Moevi et al [5] a retrouvé un cas de bilatéralité. Nous avons eut deux cas cela s’expliquerais par la longue période d’étude (14ans) et la taille de notre série. Ces caractéristiques socio-épidémiologiques sont semblable à celle de Abalo et al. [6] données qui s’accordent parfaitement aux conditions sociales du contexte Africain. L’atteinte articulaire était présente au sein de notre série à 44% des accidents. Ce résultat se rapproche de celui d’abalo et al. (51,16%) [6] par contre les travaux d’Admason et al [7], et Pietu [8] montrent des taux respectifs de 32% et 28%. Une telle implication articulaire demontre la forte exposition du genou surtout dans notre contexte où les accidents de motos sont les principaux pourvoyeurs de cette lésion. L’ouverture d’au moins un des foyers de fracture a été retrouvé au sein de 75% de genoux flottants. Ce taux est inférieur à celui de Hans Moevi et al [5] qui avait 84,5% résultats qui dépasse largement ceux de Elmrini A et al [9], et de Vieth 69% [10] la différence significative de ces résultats reflètent l’ampleur du choc au niveau du membre traumatisé dans notre contexte où les engins à deux sont devenu le mode de transport le plus fréquent. Les régions anatomiques tête et cou, suivi des membres (squelette appendiculaire) représentent près de 60% des lésions associées, très peu de travaux dans la littérature s’attardent sur cet aspect. Néanmoins, Hans Moevi [11] retrouve la même topographie. Et précise que cette association lésionnelle augmente le risque de complication pulmonaire du fait du traumatisme crânien. En comparant avec les séries de la littérature les régions topographiques qui ont des difficultés dans la prise en charge et ont nécessité la participation de plusieurs équipes sont : la région tête et cou, le thorax, l’abdomen. Car réalisant un tableau de polytraumatisme avec des scores d’ISS très élevé. Dans notre série 90% des patients était concerné par cette entité. Ce résultat contraste avec ceux obtenu par Zrig et al [12] et [13], mais reflète la sévérité des lésions concomitantes au genou flottant dans un contexte où la sécurité routière le non respect du code de la route et les excès de vitesse sont usuels.

## Conclusion

Les traumatismes crâniens, les fractures ouvertes des membres inférieurs sont de lésions concomitantes les plus fréquentes. Par ailleurs les lésions thoracique et abdominale bien que moins fréquente, réalisent des tableaux de polytraumatisme nécessitant une réanimation correcte et approprié. Ce qui explique la sévérité des lésions concomitante.

### Etat des connaissances actuelles sur le sujet

- Les travaux menés sur le sujet nous renseignent sur les aspects épidémiologiques, à savoir l’âge, le profil des patients et surtout les mécanismes conduisant à cette lésion;
- D'autres parts, plusieurs méthodes de traitements ont été ébauchées surtout dans un contexte Africain dépourvu de tous les systèmes de prise en charge des patients.

### Contribution de notre étude a la connaissance

- La particularité de notre étude est le fait que les lésions concomitantes vont largement engager le pronostique vital et fonctionnel du patient en terme de morbidité et de mortalité;
- La prise en charge oblige l’adhésion de plusieurs équipes et de spécialités différentes. Car il s’agit de véritables polytraumatisés;
- Notre étude remet en question les différentes classifications proposées jusqu’ici car ces classifications ne prennent pas en compte les lésions ouvertes. Tandis que notre étude ressort un taux considérable des lésions ouvertes des membres dont la gravité et le pronostique dépassent largement ceux de ces classifications.
